# Proton radiation effects on carrier transport in diamond radiation detectors

**DOI:** 10.1063/1.5130768

**Published:** 2020-02-03

**Authors:** Mengnan Zou, Jen Bohon, John Smedley, James Distel, Kyle Schmitt, Ren-Yuan Zhu, Liyuan Zhang, Erik M. Muller

**Affiliations:** 1 Materials Science and Chemical Engineering, Stony Brook University, Stony Brook, New York 11794, USA; 2 Accelerator Development Program Office, Los Alamos National Laboratory, Los Alamos, New Mexico 87545, USA; 3 Accelerator Operations and Technology Division, Los Alamos National Laboratory, Los Alamos, New Mexico 87545, USA; 4 Space Science and Application Group, Los Alamos National Laboratory, Los Alamos, New Mexico 87545, USA; 5 Physics, Mathematics and Astronomy Division, California Institute of Technology, Pasadena, California 91125, USA

## Abstract

Diamond, a highly radiation-resistant material, is considered a nearly ideal material for radiation detection, particularly in high-energy physics. In this study, radiation damage from high-energy proton beams was induced in diamond crystals to determine exposure lifetime in detectors made from this material; the effects were investigated using non-destructive x-ray techniques and through the FLUKA simulation package. Two diamond detectors were irradiated by an 800 MeV proton beam at different fluence rates, and the real-time current response was recorded to observe degradation in the signal over time. It was determined that the proton fluence *rate* had a significant effect on the device degradation. The detector performance from the irradiated detectors was characterized using x-ray beam-induced current measurements, and the mechanism of proton radiation damage to diamond sensors, especially the radiation effects on carrier transport, was studied. The vacancies generated from proton irradiation were considered the major source of detector degradation by trapping holes and inducing an internal electric field. Simulation results from the FLUKA package revealed an uneven distribution of the radiation-induced vacancies along the beam path, and the corresponding detector signals calculated from the simulation results displayed a good match to the experimental results.

## I. INTRODUCTION

As proton radiation therapy for cancer treatment continues to develop, an improved radiation-resistant dosimeter is required for accurate flux, position, and temporal monitoring. Diamond, due to its radiation hardness, has been employed in high-energy particle physics for a long time. Considering the other unique properties of diamond such as wide bandgap, high carrier mobility, and low intrinsic carrier density, sensors made of diamond should perform well for medical dosimetry with low leakage and rapid response. Since 1994, the RD42 group has studied the performance of CVD diamond detectors under high-energy particle beam irradiation.^1^ They observed that diamond detectors maintain constant behavior under exposure to 500 MeV proton beams with a maximum fluence rate of 10^8^ p/cm^2^/s. The proton-induced current was linear with injected proton fluence^2^ up to 10^14^ p/cm^2^, thereby establishing diamond detectors as a promising proton monitoring device for clinical applications.

One limitation of detectors for monitoring higher-flux proton beams is that these beams introduce severe damage to the detectors, leading to an obvious decay in average signal with applied proton dose.^3^ This phenomenon has been attributed to defects introduced from radiation damage, which act as trapping centers for carriers.^4^ Both electrons and holes are considered to be trapped equally. A polarization effect (asymmetric positive and negative signals) has also been observed after 24 GeV proton irradiation.^5^ The difference in polarity was explored by Grilj,^6^ who suggested the existence of an internal electric field after proton irradiation, generated from a pronounced trapping of electrons in the irradiated region. Models have been created to describe the extent of damage from proton irradiation. For instance, the RD42 group parameterized radiation damage by the lifetime of charge carriers.^7^ The reciprocal of the lifetime is linear to the integrated flux, and the damage constant k_λ_ is calibrated for proton beams with different kinetic energies.

In the previous studies, the performance of irradiated diamond detectors was only measured using the ion beam induced current (IBIC). To evaluate the potential radiation damage from ion beam exposure, we collected the photocurrent of diamond detectors using non-destructive x rays. In this paper, we monitored the detector behavior in the 800 MeV proton beam and characterized the detector response to non-destructive x rays after irradiation. The irradiation damage to the diamond detector was simulated by FLUKA, and the results fit the experimental data well.

## II. MATERIALS AND EXPERIMENTS

### A. Diamond detectors

**FIG. 1.**
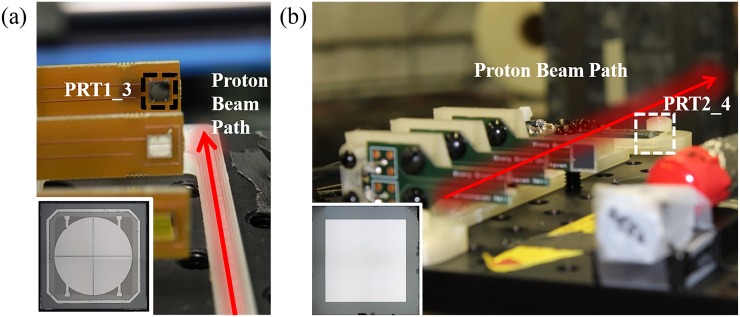
Illustration of the proton radiation experimental setups for (a) PRT1_3 and (b) PRT 2_4. Other detectors in the picture aligned along the proton beam path (indicated by the red arrow) were used for other measurements. The patterns of the Pt electrodes for PRT1_3 and PRT2_4 are shown in the insets.

Several diamonds of similar thickness were tested; we will present the detailed analysis of two representative detectors, labeled PRT1_3 and PRT2_4, which were irradiated by a proton beam in two separate runs. For these runs, up to four detectors were placed in the beam in a stacked arrangement and irradiated simultaneously. Therefore, the label represents “***P****roton* ***R****adiation* ***T****est*,” with the first number in the label indicating the run number and the second number indicating the order of the detector in the row. Both diamonds used for detectors PRT1_3 and PRT2_4 are electronic grade (nitrogen concentration < 5 ppb) single crystal CVD grown diamonds (4 mm × 4 mm) from Element Six, with platinum electrodes on both sides patterned by photolithography. To pattern the 500 *μ*m-thick diamond for the PRT1_3 detector, the electrode on the back side was a single pad used for the bias supply, while the front side was a 3 mm-diameter circular pad divided into four channels separated by 20 *μ*m-wide cross-streets in the center [inset in Fig. 1(a)]. The diamond was mounted over a 3-mm circular opening on a circuit board, which allows for subsequent transmission mode x-ray characterization. The electrode for both sides of the 300 *μ*m-thick diamond for the PRT2_4 detector was patterned as a solid 3 mm × 3 mm square [inset in Fig. 1(b)], and the diamond was hanging over the edge of the circuit board. To assemble the detectors, the diamonds were attached to the circuit board using conductive epoxy on the back side, and the front side was wire-bonded using 25 *μ*m-diameter aluminum wires for signal collection.

### B. Proton irradiation experiments

The entire area of the diamond detectors was exposed to a proton beam at the Weapons Neutron Research Facility at Los Alamos National Laboratory, with a kinetic energy of 800 MeV and a frequency of 10 Hz. A 0.6 m-thick lead shutter was installed between the sample and the source as a rapid stop of irradiation to control the incident dose. Two test runs were performed using proton beams with different sizes and fluxes.

In the first run, which had a total of three detectors, the PRT1_3 detector was the third and most downstream [Fig. 1(a)]. All detectors were aligned to the center of the 25 mm-diameter proton beam, with the larger surface perpendicular to the direction of the incident beam. Since the beam was much larger in area than the dimensions of the detector, no spatial resolution was needed; thus, the four channels of the PRT1_3 detector were bonded together for signal collection. A total fluence of 2.32 × 10^14^ p/cm^2^ was delivered to the PRT1_3 detector with an ultra-high fluence rate of 1 × 10^11^ p/cm^2^/s, with an aim to create extensive radiation damage to the PRT1_3 detector for further study.

In the second run, which had a total of four detectors, the PRT2_4 detector was the fourth detector in the detector array [Fig. 1(b)], aligned to the center of the beam by a rough line scan (electric signal maximization with optical bench horizontal motion). Detector PRT2_4 was mounted differently, with the larger surface parallel to the direction of the incident proton beam. The proton beam size was adjusted to 9 mm, and the fluence rate was changed by controlling the number of pulses passed per microsecond. A high fluence rate of 6.25 × 10^9^ p/cm^2^/s (lower than the ultra-high fluence rate of the first run) was initially used to evaluate the detector response to the proton beam. Afterwards, the PRT2_4 detector was irradiated extensively to introduce radiation damage. A 10 min-long exposure was performed using the ultra-high fluence rate beam, and then, the detector response was evaluated again using the high fluence rate beam, making the total fluence received by the PRT2_4 detector 1.54 × 10^14^ p/cm^2^.

The ion beam induced current (IBIC) from the detectors was monitored in real-time during beam exposure using Keithley electrometers. The applied electric field was maintained at 0.1 V/*μ*m for the PRT1_3 detector during the full experimental period, with a leakage current lower than 0.1 pA prior to irradiation. For the PRT2_4 detector, the highest electric field used for the test was 0.33 V/*μ*m and the leakage current with the beam off was 10 pA.

### C. FLUKA simulations

The radiation damage introduced to the detectors was simulated by the 2011.02 version of FLUKA,^8,9^ which characterizes the extent of damage by non-ionizing energy loss (NIEL) and displacement per atom (DPA).^10^ In FLUKA, the PRECISIOn mode was employed, which enables electromagnetic interactions and inelastic scattering, as well as low-energy neutrons and heavy fragment transport. The proton beam was defined as a 12.5 mm-diameter round beam with a uniform flux distribution. The dimensions of the simulated diamonds matched the experimental radiation conditions. To calculate DPA results, the displacement energy threshold used for the simulations was 43.3 eV^11^ and the atomic density was calculated as 1.76 × 10^23^ atoms/cm^3^.

### D. Non-destructive x-ray performance testing

After the proton irradiation, the detectors were characterized using non-destructive x rays at the National Synchrotron Light Source II (NSLS-II) at Brookhaven National Laboratory (Upton, NY). Different beamlines were used to test the detector performance. The beamline setup for tests is described below.

The performance of the PRT1_3 detector after irradiation was tested using a monochromatic x-ray source (15 keV) from the Inner Shell Spectroscopy (ISS) beamline at the NSLS-II. The incident beam size was defined by a 25 *μ*m-diameter aperture, and the detector was tested in a nitrogen atmosphere to avoid corrosion due to x-ray induced ozone production in the air. A high-accuracy two-dimensional motorized stage was used for raster scanning the detector, which enables mobility in the plane normal to the x-ray beam direction. The PRT1_3 detector was mounted with the quadrant surface parallel to the incident x-ray beam and facing up, while the x-ray beam induced current (XBIC) was collected from the quadrant channels separately. Two aligned x-ray beam flux monitors (XBFMs) were installed along the beam path: a nitrogen-filled ion chamber biased at 1500 V to measure the incident flux for the PRT1_3 detector and a calibrated diamond x-ray detector to measure the exit flux.

The performance of the PRT2_4 detector after irradiation was tested using the white x-ray beam from the X-ray Footprinting (XFP) of Biological Materials beamline at the NSLS-II. The incident beam size was defined by a 100 *μ*m-diameter aperture. Because of the high available flux at XFP, aluminum attenuators were employed to control the incident flux and a nitrogen atmosphere was employed to protect the detectors. The detector was mounted perpendicular to the incident beam, and two aligned XBFMs were mounted for flux monitoring: a nitrogen-filled ion chamber at 1400 V as the incident XBFM and a copper calorimeter as the exit XBFM.

## III. RESULTS AND DISCUSSION

### A. IBIC performance during proton irradiation

**FIG. 2.**
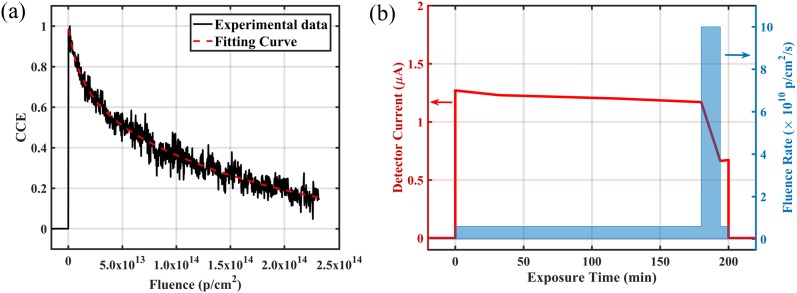
(a) CCE from the PRT1_3 detector with respect to the integrated proton fluence and corresponding fitted curve by Eq. (1) and (b) the real-time IBIC from the PRT2_4 detector monitored with exposure time, under proton beams with different fluence rates.

The PRT1_3 diamond detector was exposed to the proton beam with the ultra-high fluence rate for radiation damage evaluation. An electric field of +0.1 V/*μ*m was applied to the back-side electrode, and the IBIC signal from the front side was monitored as a function of exposure time. The carriers are generated through the entire diamond bulk from the proton beam so that the IBIC signal reflects the charge collection efficiency (CCE) from the entire detector. The relationship between the CCE and the integrated fluence is shown in Fig. 2(a), displaying an obvious decay commencing immediately following injection of the proton beam. In the previous studies,^2^ the IBIC signal from diamond radiation detectors displayed no decay with radiation up to 10^14^ p/cm^2^. However, in our case, proton beams with an ultra-high fluence rate were employed so that the detector response started decaying immediately and a fluence of 2.32 × 10^14^ p/cm^2^ already caused a dramatic change in CCE.

To describe the ultra-high fluence-rate proton-induced degradation as a function of fluence, a hyperbolic fit function [Eq. (1)] to the data is employed,^12^ where *k*_1_ and *k*_2_ are the damage parameters, ϕ is the proton fluence, and CCE_0_ is the virgin CCE before irradiation,

$$\text{CCE}\left(\phi \right)=\frac{CCE_{1}}{1+k_{1}\phi }+\frac{CCE_{2}}{1+k_{2}\phi }\;\left(CCE_{0}=CCE_{1}+CCE_{2}=1\right).$$ (1)

The data fit to two charge collection efficiencies, CCE_1_ and CCE_2_, indicating that there are two damage rates in the material similar to what is observed by Bhattacharya *et al.* In this case, CCE_1_ represents the primary damage from the incident protons and CCE_2_ is the damage caused from the cascade effect, which is the mainly affected by the fluence rate. The response of diamond detector PRT2_4 to the proton beams with different fluence rates was investigated by real-time IBIC [Fig. 2(b)]. Although two fluence rates were used during the test (6.25 × 10^9^ p/cm^2^/s and 1 × 10^11^ p/cm^2^/s), the total fluence was controlled to be nearly the same (∼7 × 10^13^ p/cm^2^) by adjusting the irradiation time. The response after the long irradiation at a high fluence rate displayed a slight decrease to 92.1%, while the response after a short irradiation with an ultra-high fluence rate decreased to 56.7%. This significant decrease was confirmed by measuring the detector response to x rays, yielding 60% of its pre-irradiation value. This indicates that the detector lifetime is not only related to the total proton fluence but is also influenced significantly by the fluence rate. The radiation damage, considered to be atomic displacements from the collision with protons, has a shorter time to self-anneal^6^ when caused by a more intense beam with a higher fluence rate. This damage, which is unable to heal before another collision, results in permanent damage that acts as a local carrier trap. Some previous studies also observed an immediate decrease in detector response under proton irradiation.^13,14^ These results reveal a non-negligible impact of the proton beam fluence rate on detector lifetime.

### B. Simulation results from FLUKA

**FIG. 3.**
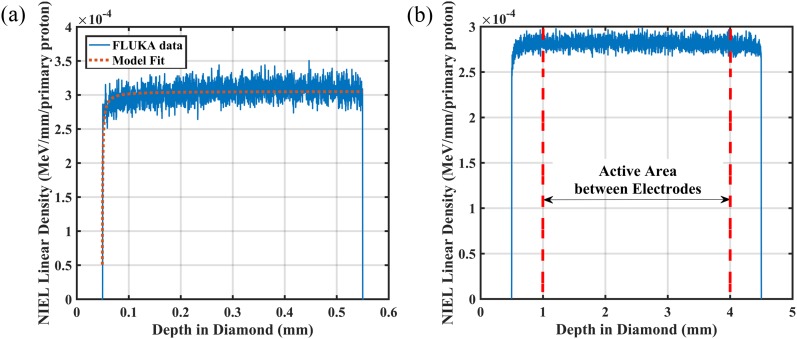
NIEL distribution from the FLUKA simulation for (a) the PRT1_3 detector with a fitting curve and (b) the PRT2_4 detector with indication for active areas between electrodes on the surface.

The distribution curves of NIEL in the diamonds are shown in Fig. 3, representing the radiation damage introduced to the PRT1_3 and PRT2_4 detectors. All the simulation parameters were kept the same, except for the dimensions of the diamonds in the proton beam. From the simulation results, the NIEL distribution indicates an increasing trend in the first 50 *μ*m in the diamond and remains flat with a slight increasing slope through the remaining depth of diamond. The DPA distribution displays a similar shape to the NIEL distribution, with a less noticeable trend. This indicates that not all NIEL events create Frenkel pairs; some energy is released by phonon generation or annihilation of Frenkel pairs (interstitial and vacancy). However, this effect is small enough that focusing on NIEL is appropriate for radiation damage evaluation. The distribution of NIEL varying with depth *x* (mm) can be fitted by the following equation:

$$\text{NIEL}\left(x\right)=\frac{b_{1}}{x+a_{1}}+\frac{b_{2}}{x+a_{2}}=\frac{{\text{A}}_{2}x+{\text{B}}_{2}}{x^{2}+{\text{A}}_{1}x+{\text{B}}_{1}}.$$ (2)

The fitting curve is plotted in Fig. 3(a), showing a good correspondence.

The DPA/NIEL distribution indicates that a cascade process has occurred in the diamond and recoiled carbon atoms were acting as knock-on atoms, also generating lattice damage in the diamond crystal. The recoiled atoms generated will have a maximum energy of 200 MeV and will stop at around 50 *μ*m, which leads to an increasing gradient in the first 50 *μ*m of diamond and a relatively flat distribution for the remaining part. The total number of vacancies introduced in the diamond is calculated to be 0.48 per primary proton, which agrees with previous results.^10,11^

### C. XBIC performance using synchrotron x-rays

As discussed above, the radiation damage from the proton beam leads to displacement of atoms by primary knock-on and cascade processes, leaving vacancies in the diamond crystals as carrier traps.^15,16^ This causes dramatic degradation in the detector response, as shown in the real-time current collected from PRT1_3 in the proton irradiation test. The density of mobile carriers for signal collection is tightly related to the density of vacancies, which is not evenly distributed in the diamonds, as simulated by FLUKA. In order to evaluate the damage along the proton beam path, a diamond crystal with a surface area of 4 mm × 4 mm needs to be irradiated with the large surface parallel to the proton beam direction such that the corresponding radiation damage at different depths will be reflected in the XBIC response map by raster scanning the non-destructive x-ray beams over the entire surface area. However, the electric field close to the diamond edges will start bending due to the edge effect from which the response will no longer reflect the status of local carriers. Consequently, the electrodes cannot cover the entire surface area, and the distance away from the diamond edge is defined by the thickness of the diamond substrate. The PRT2_4 detector was mounted in this way, with a surface area of 4 mm × 4 mm and an electrode area of 3.1 mm × 3.1 mm, missing the damage distribution information in the first 450 *μ*m. To understand the radiation damage that occurs in the first 450 *μ*m, the 450 *μ*m-thick PRT1_3 detector was irradiated with the large surface area perpendicular to the proton beam and was characterized with the response map using small x-ray beam raster scanning of the detector profile edge-on. More measurement setup details are described in Sec. II.

**FIG. 4.**
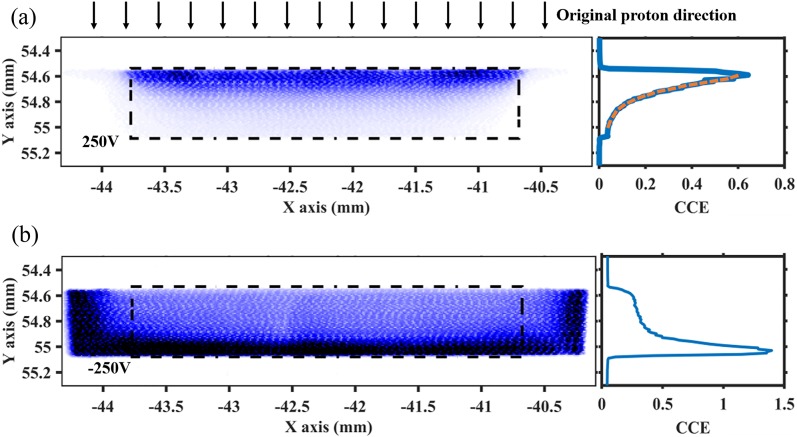
Response map from the diamond cross section of the PRT1_3 detector and corresponding average line profiles under (a) a positive (+0.5 V/*μ*m) electric field and (b) a negative (−0.5 V/*μ*m) electric field. The black frames in the response maps indicate the active area between electrodes on the surfaces, and the orange dotted line in the line profile indicates the fitting curve using Eq. (2).

A positive electric field (+0.5 V/*μ*m) was applied at the bottom side of the PRT1_3 detector, causing positive charges to move in the direction of the charge collection side, so that the collected signals reflect hole mobility. By raster scanning the entire cross section of the detector through a 100 *μ*m-diameter x-ray beam, a response map was obtained [Fig. 4(a)], which showed that the collection efficiency of holes through the depth of the diamond decreased as the distance from the collection side increased. The value of the charge collection efficiency (CCE) is determined by the actual signal collected from the detector divided by the expected signal calculated based on the known properties of diamond^17,18^ for the absorbed x-ray flux. The degradation of signal as a function of the depth in diamond was analyzed by the average line profile under the active area, reflecting a similar shape as calculated from the inverse NIEL distribution from FLUKA. Based on the linear proportion of CCE and carrier density, a similar function to Eq. (2) is applied to fit the line profile, demonstrating good correspondence with the simulation model [Fig. 4(a)].

Maintaining the same setup configuration, the applied bias was changed to reverse the electric field for the characterization of electron transport after irradiation. The response map and line profiles are shown in Fig. 4(b), displaying an opposite behavior from that observed under the positive electric field. This indicates that the number of electron traps generated from proton irradiation is very low and the electron collection is greatly influenced by the internal electric field from the trapped holes. When the x-ray beam hits the active area of the diamond, holes move toward the back side where the negative external electric field is applied, while electrons move upwards to the charge collection electrodes. Near the back side, a higher density of vacancies is able to trap more holes and, consequently, generate an internal electric field with a direction opposite to that of the external electric field. The strength of this internal electric field is proportional to the number of trapped holes and retards the electron collection. This means that when the electron–hole pairs are generated at the top side of the diamond, holes need to travel through a longer path to the back side and therefore are more likely to be trapped, resulting in a stronger internal electric field that decreases electron collection efficiency. This effect is revealed by the line profile in Fig. 4(b), displaying a decreasing CCE from the bottom to the top of the diamond with a negative applied bias. The value of CCE exceeds 1 in the line profile when the x-ray beam gets too close to the back side, which results from the backscattering from the copper layers on the circuit board.

**FIG. 5.**
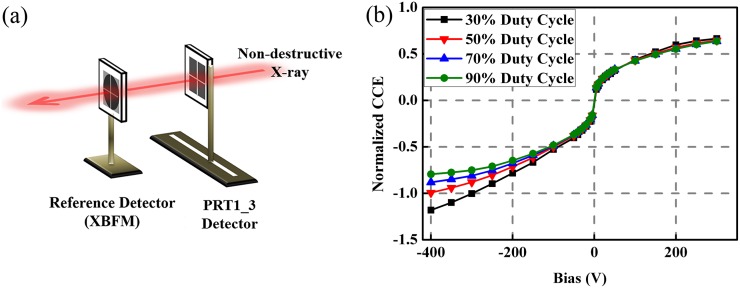
(a) Beamline setup for x-ray measurements with the PRT1_3 detector mounted with the larger surface normal to the direction of the incidence beam. (b) Current as a function of applied electric field with different duty cycles.

To eliminate the effect of the internal electric field from trapped carriers, a pulsed bias was applied for charge collection. Square wave DC bias pulses were applied with duty cycles of 30%, 50%, 70%, and 90%. During the off cycles, trapped holes are able to diffuse or to be recombined, cleaning the internal electric field from the trapped carriers.^19^ The charge collection efficiency was measured with pulsed bias of both polarities to observe the de-trapping effect on both holes and electrons. For this measurement, the diamond was remounted such that the large diamond surface was normal to the incident beam [Fig. 5(a)] and the effect from the internal electric field could be reflected directly from the CCE curve [Fig. 5(b)]. It appears that the pulsed bias may have more influence on the electron collection efficiency as compared to the hole collection efficiency. When the applied bias is negative, the direction of the external electric field is opposite to that of the internal field generated by the trapped holes such that the electron collection is retarded; the pulsed bias with low duty cycle helps to improve the charge collection efficiency. However, when the applied bias is positive, the directions of both the external and internal electric fields are the same, which may accelerate the holes, but the hole collection efficiency does not change under pulsed bias with different duty cycles.

**FIG. 6.**
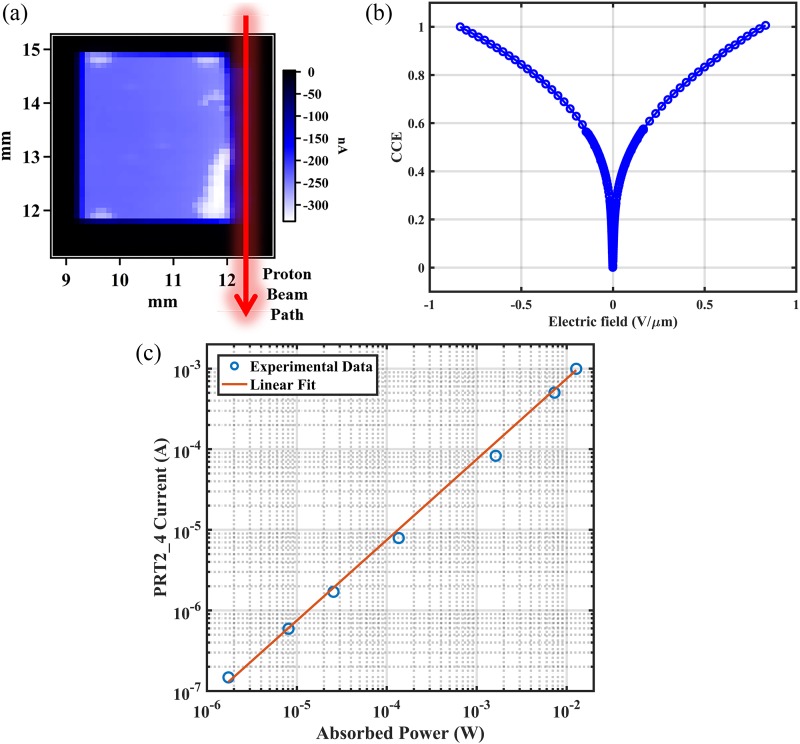
Non-destructive x-ray test results from the PRT2_4 detector, including (a) response map at −0.33 V/*μ*m, (b) CCE scan with electric field, and (c) flux scan with a linear fit. The response map demonstrated uniform response along the proton beam path direction, indicated by the red arrow. The bright regions near the edge are caused by the diamond mounting points.

As the proton beam penetrates deeper into the diamond crystal, the distribution of DPA/NIEL will be flattened to constant and no obvious slope is observed in simulation results. To prove this, the response map from the PRT2_4 detector with a negative electric field (−0.33 V/*μ*m) was obtained by raster scanning the x-ray beam (100 *μ*m-diameter) over the large surface area. The results are illustrated in Fig. 6(a), with the proton beam path direction indicated as well. Although the detector signals were below full collection calculated from the incident x-ray flux, the response over the entire active area was relatively uniform and no gradient was observed. The response maps with positive electric fields also behave similarly, which is demonstrated in the symmetric I-V curve [Fig. 6(b)]. This suggests that the radiation damage from the protons decreases charge collection efficiency and the damage distribution along the latter part of the proton beam path remains constant.

Since uniform damage was introduced in the PRT2_4 detector, the detector response should remain linear with the flux, which was verified using a 1.6 mm-diameter x-ray beam illuminating the detector center. The signals were collected by using Keithley electrometers (model 617) from the side of the PRT2_4 detector facing toward the beam, with a fixed electric field of −0.67 V/*μ*m applied to the back side. The absorbed power was calculated from the ion chamber readings and copper calorimeter temperatures, following the same method as used previously,^18^ and the corresponding detector response is plotted in Fig. 6(c). The linear relationship is maintained through the measurement (up to a flux rate of 10^14^ ph/s and an absorbed power of 0.0128 W), and the ionization energy of diamond, calculated as 13.4 eV from the inverse slope of the fitted linear slope, matches the electron–hole pair formation energy of 13.3 eV, as determined previously.^17^ This behavior indicates that the ionization energy of diamond is not changed after proton radiation and the diamond radiation detector after radiation damage could still work as a flux monitor using a high negative electric field for full collection.

## IV. CONCLUSION

The effects of proton irradiation on carrier transport in diamond radiation detectors were tested using 800 MeV proton beams of varied fluence rates. The real-time IBIC monitoring displayed significant signal degradation from proton radiation at an ultra-high fluence rate (1 × 10^11^ p/cm^2^/s), while the signal collection from irradiation at a high fluence rate (6.25 × 10^9^ p/cm^2^/s) remained stable. These results reveal that the radiation damage is not only related to the total injected dose but is also strongly influenced by the fluence rate of the incident beam. Higher fluence rates give rise to permanent atom displacements due to short self-anneal times. This permanent damage results in localized carrier traps that reduce a collection of charge carriers.

To study the signal degradation, the response from the cross-sectional side of the diamond detector was mapped and the signal was observed to change with depth. Profile maps at different polarities of the electric field reveal that the carrier transport mechanisms for holes and electrons are different. The holes are trapped by vacancies introduced by proton collision, and the distribution of vacancies is not uniform along the proton path incident to the diamond due to a cascade of recoiled carbon atoms. The FLUKA simulation of the relationship between the radiation damage (DPA/NIEL) and the penetration depth in the diamond demonstrates a rational function for the first 50 *μ*m and a stable constant deeper into the diamond. The affected signal trend along the diamond depth was confirmed by XBIC response maps from two diamond radiation detectors, and the line profiles agree with the fitted model based on the simulation results. Electron transport is also influenced by trapped holes that generate an internal electric field retarding electron collection efficiency. The diamond radiation detector with an even damage distribution displayed a linear response to incident x-ray flux, which confirms the feasibility of the devices to act as relative flux monitors even after damage.
